# Lymphangiomatosis presented with melena and chylous ascites: A case report

**DOI:** 10.1097/MD.0000000000032581

**Published:** 2023-01-13

**Authors:** Rui Nie, Jie Gao, Wei Yang, Hong Lu, Qian Ren

**Affiliations:** a The First School of Clinical Medicine, Lanzhou University, Lanzhou Gansu Province, China; b Department of Gastroenterology, the First Hospital of Lanzhou University, Lanzhou Gansu Province, China; c Key Laboratory for Gastrointestinal Diseases of Gansu Province, Lanzhou University, Lanzhou Gansu Province, China.

**Keywords:** case report, chylous ascites, enteroscopic injection sclerotherapy, gastrointestinal bleeding, lymphangiomatosis, thoracic duct obstruction

## Abstract

Lymphangioma, a rare benign tumor of the lymphatic system, is called lymphangiomatosis when it involves >1 organ, which is more rarely complicated with thoracic obstruction, the relationship between them remains unclear. With the development of enteroscopy, clinicians know more about small intestinal lymphangioma and attempt to treat it through enteroscopic injection sclerotherapy(EIS).

**Patient concerns:**

A 59-year-old male firstly manifested with gastrointestinal bleeding after a gastric perforation, who was diagnosed with lymphangiomatosis by balloon-assisted enteroscopy and abdomen CT showing >1 organ with multiple cysts besides the small intestine. The patient received an EIS, then the melena disappeared. Surprisingly he came back because of refractory ascites confirmed to be chylous by chemical tests 7 months later.

**Diagnosis:**

Lymphangiography could not determine the location of lymphatic leakage, Ultrasonography showed stenosis of the left cervical part of the thoracic duct.

**Intervention:**

On the condition that medical treatment is ineffective, thoracic duct exploration and lysis of fibrous adhesion were performed.

**Outcomes:**

Ascites significantly reduced at last.

**Lessons:**

Lymphangiomatosis is the malformation of the lymphatic system involving multiple organs, it has a possibility to be associated with thoracic obstruction. Capsule endoscopy and enteroscopy are effective methods to diagnose small intestinal lymphangioma, and EIS is an effective therapy.

## 1. Introduction

Lymphangioma is a benign tumor, it is composed of dilated, endothelial-proliferated lymphatic vessels and connective tissue. Small intestinal lymphangioma could lead to various symptoms including gastrointestinal bleeding. At present the disease was commonly treated by surgical excision, enteroscopic injection sclerotherapy is seldom used to treat it. We report a rare case of lymphangiomatosis. The patient originally presented with obscure gastrointestinal bleeding, who was treated by enteroscopic injection sclerotherapy (EIS) after being diagnosed by capsule endoscopy and balloon-assisted enteroscopy (BAE). The patient developed ascites recently, diagnosed as thoracic duct obstruction, and his symptoms were quickly relieved after surgical treatment.

## 2. Case presentation

**Figure 1.**
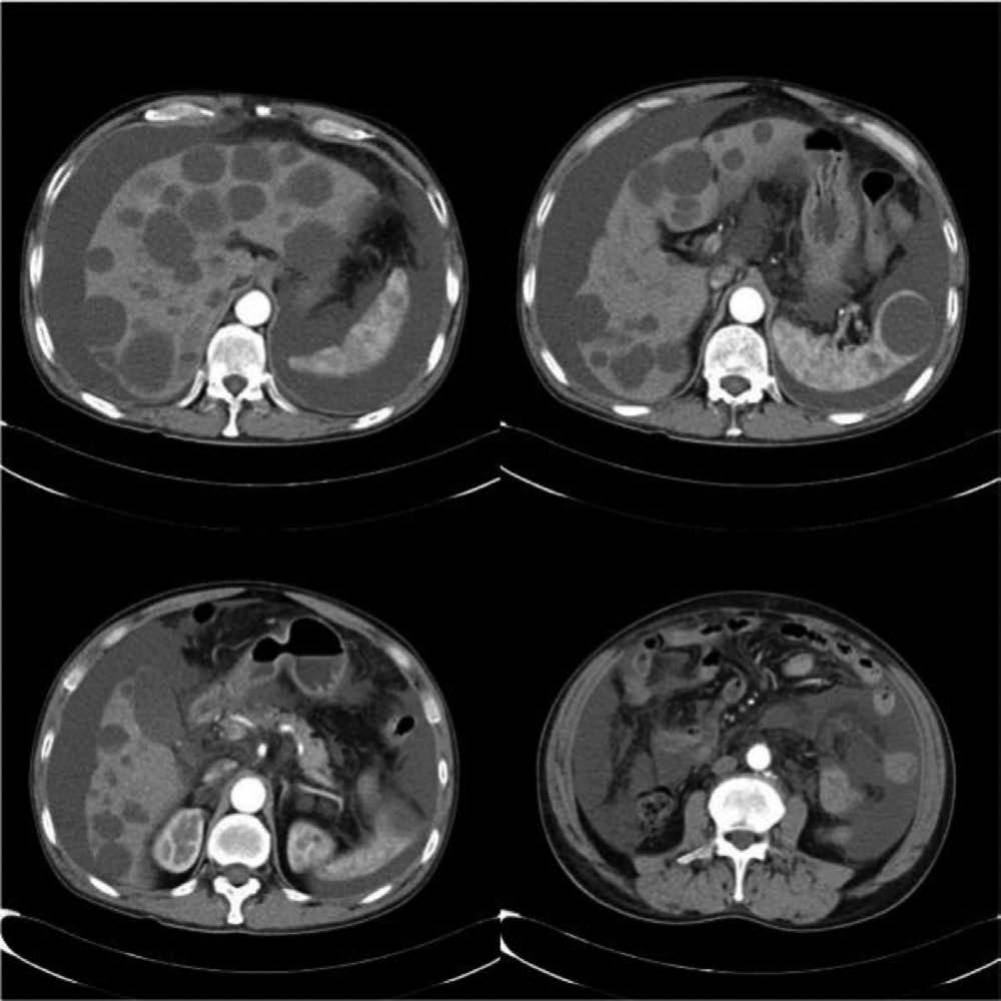
Endoscopic images of lymphangioma. Cyst-like raised lesions diffusely distributed from descending part of duodenum to jejunum. Some lesions are with hyperemia and erosion.

**Figure 2.**
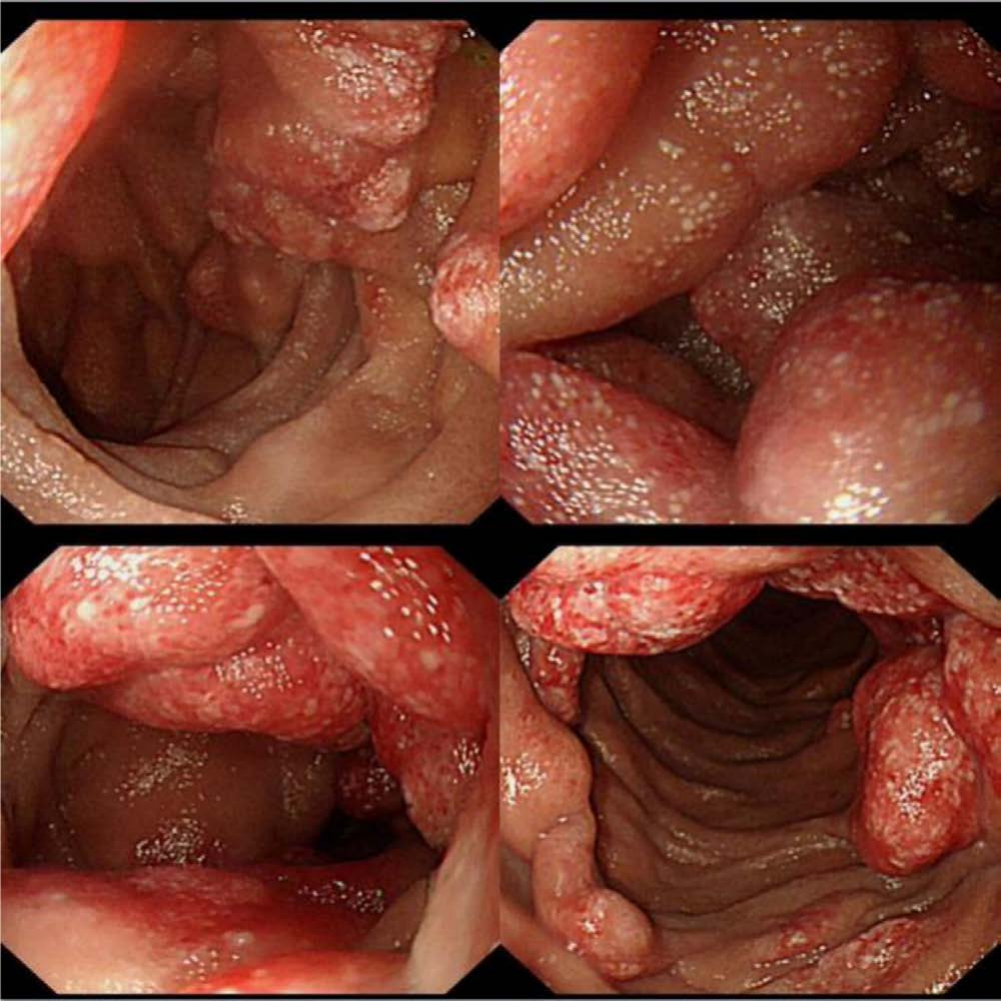
Endoscopic images of lymphangioma. Cyst-like raised lesions diffusely distributed from descending part of duodenum to jejunum. Some lesions are with hyperemia and erosion.

One and a half years ago the patient, a farmer, suddenly developed abdominal pain, he was discharged after a gastric perforation repair. But he developed recurrent melena and increasing fatigue sooner. Gastroduodenoscopy and colonoscopy did not show any bleeding lesion at the local hospital. To identify the cause of gastrointestinal bleeding the patient was transferred to our hospital. In the process of the consultation, pale lips and eyelids were distinctly observed. The laboratory values indicated microcytic hypochromic anemia with red blood cell value of 2.94 × 10^12^/L and hemoglobin value of 69g/L. Subsequently the patient was arranged to undergo a BAE. Dozens of cyst-like raised lesions with dilated lymphatics on mucosal surfaces were located from descending part of the duodenum to the jejunum (Fig. 1). Some of the raised lesions are with dilated lymphatic vessels. The range of the lesions is about 60 to 70cm. Multiple biopsies were taken, which showed chronic mucositis with dilated lymphatics in the submucosal area (Fig. 2). The immunohistochemical analysis highlighted that the endothelial cells were positive for D2 to 40.

It was interesting that the abdomen CT scan revealed multiple cystic lesions in the descending and horizontal duodenum and jejunum, multiple cysts in the mesentery root and retroperitoneum, liver, spleen, and both kidneys. Despite lacking biopsies for other organs, the patient was diagnosed with lymphangiomatosis after a discussion with multiple disciplinary doctors. Then he took oral proton-pump inhibitors, iron, and mucosal protector, which did not improve the symptoms. We advised him to transfer to the Air Force Medical Center, where the patient was treated by EIS. The doctors there determined EIS as the treatment for gastrointestinal bleeding considering the lymphangiomas were benign lesions. The bigger 56 lesions were injected with lauromacrogol from descending part of the duodenum to the jejunum, 3 of them were excised by using snares for pathological examination, and the milky liquid was seen flowing out after resection. Then gastrointestinal bleeding was cured.

**Figure 3.**
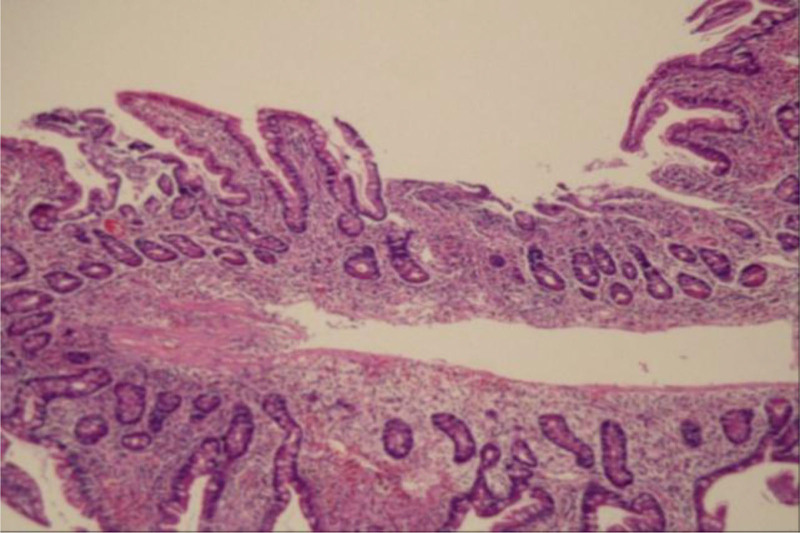
Histologic image of lymphangioma. Histology revealed chronic mucositis with dilated lymphatics in the submucosal area.

But he started to feel abdominal distension half of a year after EIS, which prompted him to go back to the hospital. Abdominal distention and shifting dullness were remarkable during physical examinations. The laboratory values indicated hypoproteinemia with albumin value of 25.7 g/L. Abdomen CT scan revealed a new finding of abdominal and pelvic effusion (Fig. 3). The volume of ascites is about 4000mL estimated through the CT images. A peritoneocentesis was performed to determine the nature and source of ascites. The patient was diagnosed with peritoneal effusion and hypoproteinemia. Examinations on extracted milky white ascites showed a high triglyceride level of 15.03mmol/L consistent with chyle. Considering the possibility that the patient had an intra-abdominal lymphangioma leak causing ascites, the patient was scheduled for a lymphangiogram, but no significant leaky site was suggested. Ascites was significantly reduced by vein nutrition and intraperitoneal catheter drainage with fluid drainage of 700 to 1000mL per day.

A month after discharge, the patient felt abdominal distension again. Then he was admitted into Beijing Shijitan Hospital, ultrasound showed lymphatic stenosis of the left cervical thoracic duct and there was a large amount of chylous fluid reflux at the venous angle. Thoracic duct exploration was performed for ascites. During the operation, it was found that the end of the thoracic duct was compressed by the fibrous tissue, resulting in the obstruction of lymphatic reflux. Then lysis of fibrous adhesion was performed.

According to follow-up 6 months after thoracic duct exploration, the patient has never complained of abdominal distension, ascites gradually reduced, and melena has never reoccurred.

## 3. Discussion

According to the histology, the classifications of lymphangioma are simple lymphangiomas, cavernous lymphangiomas, and cystic lymphangiomas.^[1]^ The etiology of the disease is still unclear. In rare cases, when it involved >1 organ or system, it is called lymphanguimatosis.^[2]^ It is generally believed that the occurrence of lymphangioma can be congenital dysplasia leading to lymphatic deposition or caused by surgery, infection, inflammation. There is another theory that some factors are expressed or overrepresented to promote the development and maintenance of lymphangioma.^[3]^ Laura J reported a patient with lymphangiomatosis was treated by Imatinib,^[4]^ which strongly supports the theory. Lymphangioma is rarely located at small intestine (<1% in all lymphangiomas).^[5]^ It is usually asymptomatic, but with the progression of the disease, a variety of clinical manifestations can occur, such as gastrointestinal hemorrhage, abdominal pain, ileus, intussusception and protein-losing enteropathy.^[6]^ Abdomen CT scan can locate and diagnose lesions, judging the number and range of lesions and the relationship between lesions and surrounding tissues.^[7]^ With the advent of capsule endoscopy (CE) and BAE, small intestinal lymphangiomas have been diagnosed more easily and accurately. We searched related articles on Chinese and Foreign databases, only 13 casesof small intestinal lymphangioma were reported in recent 5 years (Table 1). More than half of those patients presented with gastrointestinal bleeding, and the majority of lesions located at jejunum. Five patients were diagnosed by CE or BAE. Except 1 patient with a single lesion, a pedunculated jejunal polyp, was treated by endoscopic resection, other patients were treated by surgical resection. These data suggest that CE and BAE will be more and more significant tools for the diagnosis and treatment of small intestinal lymphangioma.

**Table 1:** Literature online of small intestinal lymphangioma from 2017 to 2021.

Ref	Yr	Sex	Age	Location	Symptom	Diagnostic Method	Treatment
Khan K, et al^[8]^	2017	F	24	Iluem	Intussusception	Excision biopsy	Surgical resection
Gaeta L, et al^[9]^	2018	M	54	Jejunum	Melena, anemia	Enteroscopy	Surgical resection
Wang J, et al^[10]^	2018	F	31	Jejunum	Fatigue	Capsule endoscopy and enteroscopy	Surgical resection
Lim DR, et al^[11]^	2018	M	70	Jejunum	Melena, anemia	Excision biopsy	Surgical resection
Samuelson H, et al^[12]^	2018	M	70	Jejunum	Intussusception	Excision biopsy	Surgical resection
Zhen Y, et al^[13]^	2018	F	77	Jejunum	Melena, anemia	Excision biopsy	Surgical resection
Yang J, et al^[14]^	2019	M	26	Iluem	Melena, anemia	Excision biopsy	Surgical resection
Harris E, et al^[15]^	2019	F	57	Jejunum	Ileus	Excision biopsy	Surgical resection
Giuliani A, et al^[16]^	2019	M	41	Iluem	Ileus, intestinal perforation	Excision biopsy	Surgical resection
Tan B, et al^[17]^	2020	F	29	Jejunum	Melena, anemia	Enteroscopy	Surgical resection
Mohammed AA, et al^[18]^	2020	M	31	Iluem	Ileus	Excision biopsy	Surgical resection
Wu TL, et al^[19]^	2020	F	84	Jejunum	Melena, anemia	Enteroscopy	Enteroscopic resection
Ding XL, et al^[20]^	2021	M	78	Iluem	Melena, anemia	Enteroscopy	Surgical resection

In this case, although the pathological specimens only included small intestinal lesions, we diagnosed him with lymphangiomatosis based on his clinical characteristics and abdominal CT. Unlike the majority of reported cases, this patient is complicated with thoracic duct obstruction and developed chylous ascites. It is currently believed the diseases of cervical thoracic duct reflux disorder are associated with generalized lymphatic anomaly. In a retrospective analysis of 9 patients with lymphangiomatosis including spleen, bones, axillas, peritoneum, retroperitoneum and etc, Sun Ying et al found 5 of these patients complicated with concurrent thoracic duct obstruction.^[21]^ One view is that thoracic duct obstruction and other lymphatic diseases are parts of a systemic disease featured with systemic lymphatic hyperplasia, narrowed lumen and lymphatic leakage. Another view is that thoracic duct obstruction is the primary pathological change causing lymphatic malformations in other organs.

Generalized lymphangiomas leading the blood supply deficiency of intestinal musoca caused a persistent state of chronic hypoxia and inflammation. Under stress caused by surgery, due to the excitation of sympathetic nervous system, intestinal mucosal blood vessels are strongly contracted and blood perfusion is reduced, which can aggravate the damage to intestinal mucosa. We believed it is the reason why gastrointestinal bleeding occurred after the gastric perforation repair. When lymphatic malformations are generalized and persistent, the high pressure of the lymphatic system inevitably builds up. We hypothesized that the cause of ascites was a sharp aggravation in the high pressure of the lymphatic system caused by EIS.

The common treatment of lymphangioma still is surgery, which can completely remove the lesions especially if they are large or diffuse. Presently physicians prefer a less invasive treatment because lymphangioma is a benign disease. EIS is mostly used in the therapy of vascular malformation. In this case, although the range of lesions was about 70cm long, gastrointestinal bleeding and anemia were cured through EIS. It supports that EIS is a feasible and effective method to treat small intestinal lymphangioma.

## 4. Conclusion

In summary, we report a case of lymphangiomatosis diagnosed by enhanced abdomen CT scan, CE and BAE, which suffered from gastrointestinal bleeding treated by EIS and successively chylous ascites treated by thoracic duct lysis of fibrous adhesion. Lymphangiomatosis is a disease of generalized lymphatic anomaly, and as yet little is known of the causes of the disease. So more further studies about its etiology, clinical features, diagnosis and treatment are significant. When it affects the small intestine, it can cause 1or more gastrointestinal symptoms. It is necessary that this disease should be under consideration in this situation. CE can be the preferred inspection method for screening, and BAE combined with pathological biopsy can be performed for precise diagnosis. EIS is an available treatment of small intestinal lymphangioma. If CT scan reveal cystic lesions in mutiple organs and the lesions are thought to be caused by malformation of the lymphatic system, it is necessary to perform a lymphangiography to know about the specific situation of lymphatic malformation. It is not necessary to treat the asymptomatic patients, but essential to monitor for potential complications through physical examination, imaging, or laboratory test in follow-up period.

## Author contributions

**Conceptualization:**Rui Nie, Qian Ren.

**Funding acquisition:**Wei Yang,Qian Ren.

**Supervision:**Qian Ren.

**Writing – original draft:**Rui Nie,Jie Gao.

**Writing – review & editing:**Wei Yang,Hong Lu.
